# Designing a Small Molecule for PET Radiotracing: [^18^F]MC225 in Human Trials for Early Diagnosis in CNS Pathologies

**DOI:** 10.3390/molecules30183696

**Published:** 2025-09-11

**Authors:** Francesco Mastropasqua, Gert Luurtsema, Cristina Filosa, Nicola Antonio Colabufo

**Affiliations:** 1 Dipartimento di Farmacia-Scienze del Farmaco, Università Degli Studi di Bari ALDO MORO, Via Orabona 4, 70125 Bari, Italy; nicolaantonio.colabufo@uniba.it; 2 Department of Nuclear Medicine and Molecular Imaging, University Medical Center Groningen (UMCG), University of Groningen, 9712 CP Groningen, The Netherlands; g.luurtsema@umcg.nl; 3 Biofordrug, Spin-Off of the University of Bari, Via Dante 95/99, 70019 Bari, Italy

**Keywords:** P-glycoprotein, tracers, PET

## Abstract

P-Glycoprotein (P-gp, also known as MDR1 or ABCB1) is an ATP-binding cassette (ABC) transporter that actively effluxes a wide range of structurally and functionally diverse molecules, playing a crucial role in drug absorption, distribution, and excretion. P-gp is highly expressed at key biological barriers, such as the blood–brain barrier (BBB), intestine, liver, and kidneys, and it serves as a gatekeeper against xenobiotics and therapeutics. Its dysregulation is involved in multidrug resistance (MDR), epilepsy, cancer, infectious diseases, and neurodegenerative disorders. Several small molecules were synthesized using SAfIR and SAR, and, among them, [^18^F]MC225 showed the most promising results for in vivo human studies, with appropriate pharmacodynamics and pharmacokinetics profiles for in vivo use. [^18^F]MC225 is currently being employed in PHASE II human trials at the UMC Groningen, the Netherlands, in patients diagnosed with AD, PD and MCI, as well as PHASE II human trials at the Policlinico Gemelli in Rome Italy to diagnose P-gp resistant depression. Preliminary studies show that [^18^F]MC225 radiotracer is behaving according to the initial predictions, that is, it accurately diagnoses the aforementioned pathologies, more so than previously developed small molecules for the same goal.

## 1. Introduction

**Figure 1:**
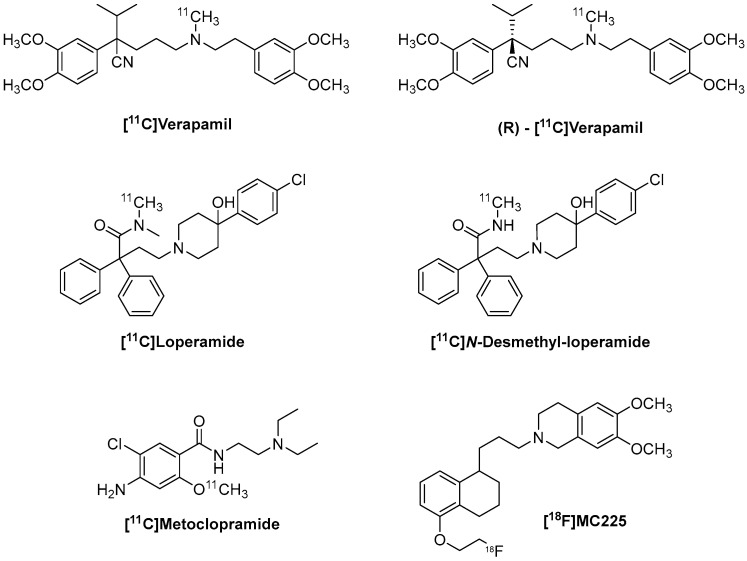
Structures of the main radiotracers.

P-Glycoprotein (P-gp), an ATP-binding cassette transporter (ABC), is a membrane-bound efflux pump that utilizes energy derived from ATP hydrolysis to transport a wide variety of unrelated molecules out of cells, despite their concentration gradient, playing a crucial role in cellular drug absorption and excretion [1,2]. It is a glycoprotein of approximately 170 kDa encoded by the multidrug resistance gene-1 (*mdr1*), highly expressed in various organs, particularly in the luminal membrane of the small intestine and the blood–brain barrier (BBB), as well as in the apical membranes of excretory cells such as hepatocytes and renal cells [3]. Changes in P-gp expression or function could lead to several diseases, both in the central nervous system (CNS) and in chemo-resistant morbidities [4]. Throughout this review, we will analyze P-gp in detail in terms of structure, localization, function, regulation, and mechanism of action. In particular, we will focus on studying P-gp function through Positron Emission Tomography (PET), emphasizing the main radiotracers currently used, such as [^11^C]Verapamil and (R)-[^11^C]Verapamil, [^11^C]Loperamide and [^11^C]-*N*-desmethyl-loperamide, and [^11^C]Metoclopramide and [^18^F]MC225 (Figure 1), a radiotracer developed in an academic setting and currently in Phase II clinical trials. The aim of this review is to provide insight into how the application of these radiotracers for measuring P-gp function and visualization has changed over the years, especially regarding CNS pathologies, highlighting the differences between preclinical and clinical settings.

## 2. Localization, Structure, and P-gp Activity

### 2.1. Localization

P-gp shows high expression on the apical surface of the epithelium of intestinal cells, liver bile ducts, and proximal renal tubules. Since all these organs have an excretory role, it was suggested that the pump exerts its physiological role in the elimination of endogenous or xenobiotic metabolites through Phase III-mediated transport. Additionally, it makes the membrane less permeable to drug passage. P-gp is localized in several biological barriers at the apical levels of endothelial cells, both in CNS and in other organs where the flow/transit of xenobiotics needs modulation. This protective role of P-gp is strongly supported by studies performed with knockout transgenic mice lacking one or both genes encoding P-gp transporting drug, Abcb1a and Abcb1b [5,6]. At the intestinal level, P-gp is highly expressed in the epithelial cells of the colon and ileum (apical side), while in the jejunum, duodenum, and stomach, it is relatively reduced compared to the ileum. Here, it plays a key role in drug absorption as it expels the drug and limits its oral bioavailability, reducing its effectiveness in vivo. This is a very important aspect to consider because among naturally lipophilic drugs (about 50–60% of new chemical entities and 40–50% of existing ones), it is estimated that more than 25% are P-gp substrates [7,8]. An interesting aspect of P-gp expression in the small intestine is its interaction with metabolizing enzymes, particularly with CYP3A4, a cytochrome P450 isoenzyme, towards which there is considerable overlap in substrate specificity and tissue distribution [9]. P-gp is also expressed throughout the respiratory tract where it plays an important role in protection against the entry of endogenous or exogenous toxic compounds into the lungs. In the human lung, P-gp is expressed on the apical side of ciliated epithelial cells, ciliated and apical collecting ducts, and lateral surfaces of serous cells of bronchial glands but not in mucin-secreting goblet cells. Mutations in the MDR1 gene affect the natural protection of the respiratory tract by accumulating toxic compounds; however, if P-gp is overexpressed, xenobiotics are metabolized quickly and expelled from the cells, which is particularly important for the treatment of lung cancer. Several studies demonstrated that a number of drugs, usually employed in the treatment of chronic obstructive pulmonary disease (COPD), are P-gp substrates. Following this finding, the concentration of said drugs is lower than expected, and this supports the fact that P-gp can limit the pulmonary absorption of P-gp substrates [10,11,12]. P-gp is also present in placental tissue of various mammalian species, including humans. It localizes on the apical microvillous surface of syncytiotrophoblasts (STBs), where it participates in the efflux of hydrophobic and cationic drugs into the maternal blood, thus limiting fetal exposure to drugs and xenobiotics. The expression of placental P-gp during pregnancy is not uniform; in mice and rats, P-gp expression reaches its peak during the second half of pregnancy and then decreases towards term; however, in humans, a decrease in placental expression is observed as gestation progresses. Therefore, placental P-gp seems to be upregulated in the early Phase of human pregnancy, probably to protect the fetus from potentially toxic xenobiotics. Fetal protection has been confirmed by numerous in vivo experiments using genetically modified mice or using in situ perfusion of rat placenta [9,13,14]. Furthermore, it is commonly accepted that P-gp inhibition can affect drug targeting by enhancing the drug toxicity of drugs such as HIV protease inhibitors and antitumor chemotherapeutic agents. Therefore, Eyal S. et al. conducted an experiment on pregnant macaques using [^11^C]Verapamil as a model substrate of P-gp and Cyclosporin A (CsA) as an inhibitor, testing the feasibility of using non-invasive and simultaneous PET to evaluate P-gp activity and inhibition in maternal organs and placenta. The experiment demonstrated that inhibition of P-gp by CsA resulted in a significant increase in the distribution of [^11^C]Verapamil radioactivity in tissues most protected by P-gp, namely, the maternal brain and fetus [15]. P-gp is also present at the cardiac level, particularly in arterioles and capillaries of the heart. This is a very important aspect to consider because several drugs employed in the treatment of heart failure are P-gp substrates; therefore, the actual drug concentration is lower than expected when P-gp is over expressed, thus reducing bioavailability, since P-gp is localized between blood and cardiomyocytes (Table 1) [16,17].

### 2.2. Structure

**Figure 2:**
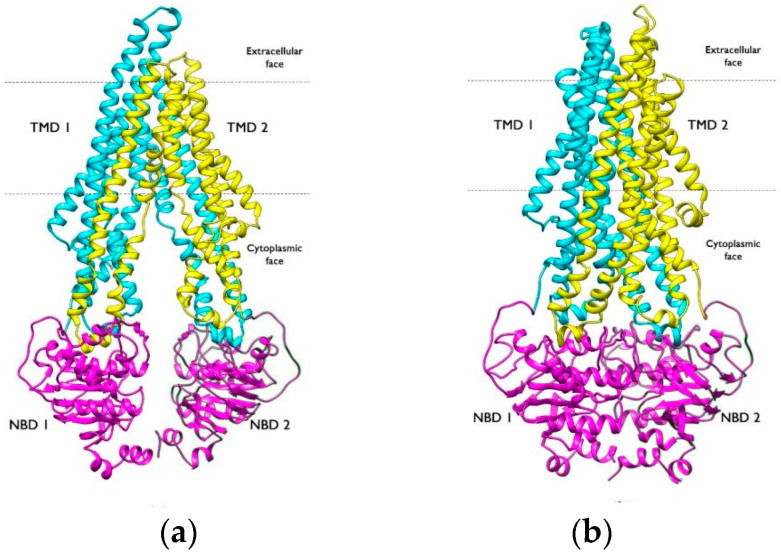
Shift in P-gp’s physiological state during its activity. Cryo EM structures of h P-gp. (**a**) Resting state of P-gp, corresponding to the inward-facing conformation (PDB ID: 6QEX); (**b**) outward-facing conformation (PDB ID: 6C0V). Nucleotide-binding domains (NBDs) are shown in magenta, and transmembrane domains (TMDs) are shown in cyan and yellow [20].

P-gp is a plasma membrane protein organized into a single polypeptide of 1280 residues with a molecular weight of 170 kDa. It contains two transmembrane domains (TMDs) consisting of 12 transmembrane α-helices each and two nucleotide-binding cytosolic regions (NBD) that are highly expressed when water is involved in ATP hydrolysis; these two halves are symmetric to each other and are connected by a highly charged “linker region,” approximately 75 amino acids long and phosphorylated at different sites by protein kinase C (PKC). The two halves of the molecule do not need to be covalently bound to function. The TMDs, representing the hydrophobic portion of P-gp, contain the drug-binding domains (DBSs) and create the pathway through which drug molecules cross the membrane, causing P-gp to shift from an inward-facing to an outward-facing conformation (Figure 2). The structure of P-gp transporter displays specific NDB domains, namely, Walker A e Walker B, whereas the TMDs are structurally superimposed to other ABC pumps (sister pumps) [2,5,18,19,20].

An essential aspect to consider is the flexibility of P-gp, which, like any protein in natural conditions, is flexible in an aqueous environment; specifically, it must adapt its specific dimensions to different ligands. P-gp has an unusually broad poly-specificity, allowing it to easily change the size of its internal cavity, thus recognizing hundreds of compounds. X-ray crystallography of the inward-facing conformation of P-gp has revealed the dimensions of the internal cavity (6000 Å^3^), which are sufficient to accommodate at least two compounds simultaneously. High flexibility is also provided by the tertiary structure of P-gp, allowing for three-dimensional reorientation; in fact, it was demonstrated that P-gp interacts with various stereoisomers of the same compound. In addition to that, molecules displaying an MW between 250 and 4000 Da are also P-gp substrates, especially if they present hydrophobic moieties that interact with the lipid bilayer, where P-gp binds them selectively. Owing to the main binding cavity (MBC) and other binding sites (OBSs), P-gp displays great binding activity, which leads to a pharmaceutical side-effect occurring, determining multidrug resistance (MDR) [21,22,23]. The flexible structure of P-gp is responsible for its translational and rotational movements during the efflux mechanism. Conformational changes, both local and global, are induced by the entry of a suitable efflux substrate and lead, among other things, to a simultaneous variation in NBD distance. In P-gp, intracellular coupling helices (CHs), each formed by two TMs, mediate physical connections with the NBDs. In the case of substrate binding, a series of small but significant displacements occurred, mainly in CH2 and CH3, leading to the reorientation of the NBDs towards a pre-dimer configuration. Cross-linking showed that the conformational flexibility of CH2/CH3 and the hydrophobic network at the CH2/CH3/NBD2 transmission interface were critical for P-gp transport activity. A key role in the conformational transition is played by TM4 and TM9, as conformational changes induced by the substrate in TM4 and TM9 may provide allosteric communication with the NBDs through CH2 and CH3. Based on experimental observations, the secondary structure of P-gp does not change during its catalytic function [1,24].

## 3. Role of P-gp in CNS Diseases

CNS pathologies represent a constant challenge in medicine and research as they are steadily increasing, and in most cases, therapeutic responses are poorly effective. ABC transporters play an essential role in many neurodegenerative pathologies because altered expression and functionality of these transporters have been observed in many of them. Additionally, they reduce the efficacy of many drugs since they are substrates for these transporters [25,26]. Within this class of transporters, a particularly important role is played by P-gp because it limits the penetration of many exogenous agents across the BBB and appears to be involved in the etiology of some neurological disorders. Several studies demonstrate that the function of P-gp decreases in the advanced stages of neurodegenerative diseases such as Parkinson’s and Alzheimer’s. Dysfunction of P-gp may contribute to neuronal damage induced by the increased accumulation of toxins, as seen in Parkinson’s disease (PD), or by the reduced ability of the brain to expel accumulating proteins, as in Alzheimer’s disease (AD) [27,28].

### 3.1. Role of P-gp in Alzheimer’s Disease

Neuritic plaques and neurofibrillary tangles are typical in vivo biomarkers of AD present in brain parenchyma [27,28]. In particular, the amyloid theory reports that two different peptides are accumulated in brain tissues when amyloidogenic pathways are prevalent with respect to physiological non-amyloidogenic ones. Among the numerous isoforms of beta-amyloid with subtle differences depending on the number of C-terminal amino acids, Aβ 1–42 plays a fundamental role in AD pathogenesis. The neurotoxic potential of the Aβ peptide stems from its biochemical properties that promote aggregation into insoluble oligomers and protofibrils [29,30]. The P-gp plays a critical role in endothelial efflux of Aβ; indeed, it normally transports Aβ out of the brain. A reduction in P-gp efflux function at the BBB can therefore lead to an accumulation of insoluble proteins involved in neurodegenerative diseases in the brain [31,32,33]. Several studies demonstrate that a reduction in P-gp expression in brain capillaries at the BBB decreases the clearance of Aβ from the brain into the blood, significantly contributing to the accumulation and burden of Aβ in Alzheimer’s disease [10,34]. The highest expression of P-gp has been found in the brainstem, where the lowest levels of Aβ plaques have also been detected. This discovery of the highest level of P-gp expression within the brainstem is observed in both normal brains and brain tissue of patients with Alzheimer’s disease [35]. Ding Y. et al., using a transgenic mouse model of AD (mice overexpressing the human amyloid precursor protein (hAPP), Tg2576), discovered that hAβ40 causes degradation of P-gp in brain capillaries. Specifically, hAβ40 triggers the degradation of P-gp through the activation of the ubiquitin–proteasome pathway; by preventing the ubiquitination of P-gp, the expression of P-gp protein and its transport activity at the blood–brain barrier can be preserved, leading to lower levels of Aβ in the brain in vivo [36].

### 3.2. Role of P-gp in Parkinson’s Disease

Parkinson’s disease (PD) is a chronic neurodegenerative disorder characterized by a progressive and selective loss of dopaminergic neurons in the substantia nigra (SN) and the presence of intracytoplasmic Lewy bodies (abnormal aggregates of the α-synuclein protein) within these neurons. In addition to these hallmark features of the disease, involvement of the BBB in the pathogenesis of PD has also been recognized, highlighting various changes in its structure and function [37,38]. There is a strong correlation between reduced expression of P-gp and an increased risk of Parkinson’s disease. Mice lacking P-gp show a high accumulation of pesticides and other harmful toxic substances in the brain, indicating its importance [39]. However, it has not been possible to confirm a correlation between the reduced function of P-gp at the BBB and the onset of Parkinson’s disease [40]. Several studies therefore demonstrate that the reduced efflux of P-gp is not the main cause in the onset of PD, but rather, the function of P-gp decreases with aging and in the advanced stages of various Parkinsonian diseases [41,42,43]. Bartels A.L. et al. conducted an in vivo PET study on seventeen healthy volunteers aged between 18 and 86 years old. By using [^11^C]Verapamil, it was shown that a more profound functional decrease in P-gp was observed in the regions of the white matter of the internal capsule and the corona radiata, as well as in the orbitofrontal regions [44].

### 3.3. Role of P-gp in Other Diseases

The P-gp is also involved in the etiopathology of other CNS disorders such as Huntington’s disease (HD). Kao Y.H. et al. demonstrated that the expression of P-gp is upregulated in cerebral capillaries in the cerebral cortex and striatum of human patients with HD; in particular, P-gp expression in the cortex increased with the progression of the disease, and P-gp mRNA levels were higher in a later stage of the disease [45]. The P-gp is also involved in other conditions such as Schizophrenia [46] and Major Depressive Disorder (MDD) [47,48].

## 4. P-gp and Multidrug Resistance (MDR)

Multidrug resistance (MDR) is responsible for over 90% of deaths among cancer patients receiving traditional chemotherapeutic agents or new targeted drugs [49].

MDR is the cellular mechanism through which tumor cells develop resistance to various structurally and mechanistically unrelated chemotherapeutic drugs. It can manifest through various mechanisms, with the primary one being the overexpression of P-glycoprotein [50]. It mediates MDR in many tumors such as breast cancer, lung cancer, human colorectal cancer, ovarian cancer, and others. Its expression confers high resistance to chemotherapeutic drugs such as taxanes (Paclitaxel), vinca alkaloids, anthracyclines, and camptothecins [51]. The P-gp expels a wide variety of drugs from tumor cells, leading to a decrease in intracellular drug concentration and the MDR phenotype; thus, it limits the therapeutic effect, resulting in low bioavailability [52]. Pettersson M. et al. demonstrated that drugs with a higher molecular weight have a lower risk of efflux by P-gp; specifically, efflux pumps do not affect macromolecules like proteins because they are too large to be expelled [53]. Regarding methodologies to reverse MDR, the strategy of molecular-level P-gp downregulation has proven to be less effective compared to blocking P-gp efflux function. It has been hypothesized that inhibiting P-gp efflux function could represent a potential therapeutic strategy to overcome MDR. Although it has been shown that numerous P-gp inhibitors significantly enhance the efficacy of some antitumor drugs in MDR tumor cells in vitro and in vivo, no P-gp inhibitor has been approved for clinical use [54].

## 5. PET Imaging and the Importance of P-gp

PET is a non-invasive nuclear imaging technique and it is characterized by greater tissue penetration, superior quantification, and easy translatability from animal to human. The radioligand labeled with positron-emitting radionuclides loses its energy during decay and generates radiation detected externally and measured through a “PET scanner”. PET radiotracers are characterized by robust pharmacodynamics and pharmacokinetics owing to a thorough drug discovery process [55,56,57]. Molecular imaging is a useful research tool for non-invasively visualizing and studying inflammation in vivo in a variety of diseases [58]. It also has the potential to study drug resistance mechanisms in patients, thus increasing understanding of drug action in vivo, assessing their biological effects, and enabling treatment individualization [59]. The high-performance imaging of PET is obtained with multimodal devices such as PET-CT and PET-MRI that provide both functional and anatomical data simultaneously [60,61]. To date, [^18^F]-fluorodeoxyglucose (FDG) is the most employed tracer in both oncological and neurological diseases, even though it only reflects the diseases’ metabolic activity, without considering drug–target interactions involved in the inflammatory process. An essential aspect to consider is the choice of radionuclide; in fact, several factors need to be considered, such as the decay characteristics suitable for optimal resolution and quantitative accuracy, and, in the case of immuno-PET, it should allow for easy, efficient, and stable coupling with mAbs. The measurement of P-gp function with PET has mainly been conducted with low-molecular-weight tracers where even small changes in molecular structure can cause unwanted alterations in pharmacokinetic and pharmacodynamic properties [62,63]. An accurate description of radionuclide selection criteria for radiolabeling has been provided by Serdon K. et al. [64]. The presence of carbon in natural products and pharmaceutical compounds makes carbon-11 (^11^C) an isotope that emits positrons to label molecules of biological interest. Fluorine-18 (^18^F), on the other hand, is the most commonly used radionuclide for its maximum half-life of 110 min and thus easy access to clinical PET centers. Nevertheless, producing a ^18^F radiotracer carries some disadvantages, such as the relatively small pool of biologically active fluoroorganic target molecules. In addition to that, the effects that the introduction of a fluorine atom on a molecular fragment may have on the biological properties of the newly labeled compound are unknown, and the difficulties that arise in making direct comparisons between the biological properties of the parent non-fluorinated molecule to the ^18^F-labeled counterpart are not negligible [60,65,66]. The development of a new radiotracer should take into account several aspects, such as high in vivo drug targeting, drug stability compatible with the half-life of the chosen radionuclide, and finally an adequate clearance in the targeted organs [16,67].

PET imaging of P-gp function was first demonstrated in 1998 using verapamil labeled with ^11^C. The basic idea when using labeled P-gp substrates as PET tracers is that a low brain concentration suggests efficient P-gp function, while a high concentration indicates less efficient P-gp function [62]. Many studies demonstrate that P-gp activity/expression can correlate with the early onset of not only CNS diseases, but also with other pathologies such as chemo-resistant tumors, and HIV encephalopathy [68].

By using radiolabeled P-gp substrates, the contribution of P-gp to the pharmacokinetics of drugs in vivo can also be studied. Increased brain uptake in animals or humans of the radiolabeled drug after pre-treatment of a modulator indicates drug transport mediated by P-gp. Increased brain absorption of the drug under examination in P-gp knockout mice compared to wild-type mice can also prove that the drug under examination is actively expelled from the brain [69,70,71]. Radiotracers for P-gp measurements are typically administered intravenously. Since the distribution of the PET radiotracer in the brain depends on the arterial concentration entering the brain, arterial blood is also sampled. Once the radioactivity concentration in the brain measure by the PET scan and arteries is obtained, quantification of the data can be performed using two different approaches. Calculated kinetic parameters reflect P-gp function at the BBB. These approaches are called NON-COMPARTMENTAL METHODS and COMPARTMENTAL METHODS [72].

## 6. [^11^C]Verapamil and (R)-[^11^C]Verapamil

Verapamil is a voltage-dependent calcium channel antagonist used to treat hypertension, angina pectoris, and supraventricular tachycardia. It is also the oldest tracer developed for PET imaging of P-gp in humans, and thus has been the subject of the largest number of clinical studies. Verapamil is a P-gp substrate, but at the microdoses used for PET imaging, [^11^C]Verapamil acts as a strong P-gp substrate [73,74]. [^11^C]Verapamil has been used to measure P-gp function at BBB in vivo based on the fact that its penetration into the brain reflects P-gp function, specifically measuring the decrease in P-gp function as an increase in tissue radioactivity uptake. Using this radioligand, possible changes in P-gp function have been suggested in neurological disorders such as PD, AD, or epilepsy [75,76]. [^11^C]Verapamil was initially used as a racemic mixture, but it was later discovered that enantiomerically pure (R)-[^11^C]Verapamil is preferable for the kinetic modeling of PET data due to differences in metabolism and plasma protein binding between (R)- and (S)-Verapamil [77]. Verapamil works a calcium channel blocker in both its enantiomers. Nevertheless, the *S*-enantiomer displays a much greater efficiency as a blocker, namely, around 10 times more. On the other hand, the *R* enantiomer is preferably used as a P-gp radiotracer because it acts as a stronger substrate for this protein [78]. Luurtsema G. et al. demonstrated the pharmacodynamic profiles of both Verapamil enantiomers in several animal models, showing that, despite the fact that both enantiomers are P-gp substrates, the *R* enantiomer is a safer PET tracer for measuring P-gp function in vivo [79,80].

### 6.1. Metabolism and Kinetic Evaluation of [^11^C]Verapamil and (R)-[^11^C]Verapamil

**Figure 3:**
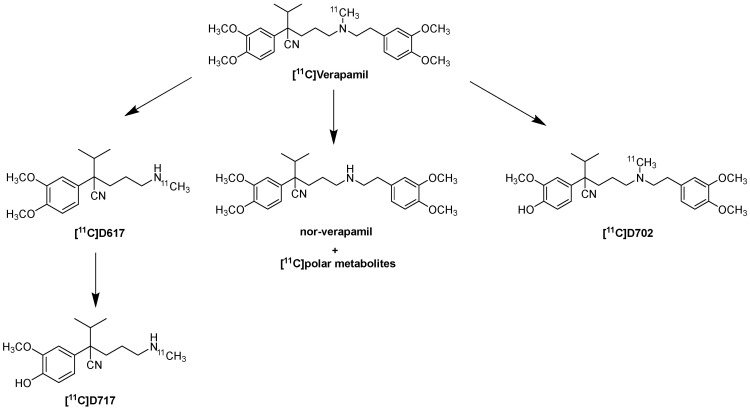
Structure of [^11^C]Verapamil and its major metabolites [81].

Verapamil’s main metabolic pathways include *N*-dealkylation (producing D-617 and D-717), *N*-demethylation (producing norverapamil and formaldehyde), and *O*-demethylation (producing D-703 and D-702). The metabolites obtained from *N*-dealkylation are [^11^C]D-617 and [^11^C]D-717, and from *O*-demethylation, [^11^C]D-702 and [^11^C]D-703, as shown in Figure 3. Additionally, small-labeled carbon fragments are formed. It is predicted that [^11^C] formaldehyde is the main metabolite, which can be further converted into many other metabolites and eventually into [^11^C]CO_2_. A schematic representation of the main metabolites is shown in Figure 3. Thus, the study by Verbeek J. et al. highlights how these [^11^C]-labeled metabolites may contribute to the measured PET signal. In particular, [^11^C]D-617 is a P-gp substrate with similar brain kinetics to (R)-[^11^C]Verapamil under basal conditions. P-gp inhibition led to a much smaller increase in brain uptake of [^11^C]D-617, suggesting a lower affinity for P-gp compared to (R)-[^11^C]verapamil. In contrast, the study conducted by Luurtsema G. et al. shows that aside from [^11^C] polar metabolites, no other [^11^C] metabolites of verapamil (such as [^11^C]D-617 or [^11^C]D-703) were detected in the rat brain. The formation of [^11^C] polar metabolites, such as formaldehyde, could lead to levels of [^11^C] in the brain that are not mediated by P-gp. This could influence the measurements of BBB P-gp function [81,82,83]. Ikoma Y. et al. conducted a quantitative analysis of [^11^C]Verapamil transfer from plasma to the brain of healthy volunteers, showing that the amount of not metabolized [^11^C]Verapamil was approximately 94% at 7 min, 83% at 12 min, 55% at 30 min, and 35% at 60 min. This study also demonstrates that the existence of metabolites modifies BBB permeation, and it suggests the possibility that metabolites contribute to the measured activity [84]. Currently, the preferred model for the kinetic analysis of (R)-[^11^C]Verapamil is a single-input model with a single tissue compartment, using the sum of (R)-[^11^C]Verapamil and *N*-dealkylation metabolites in the plasma as the input function, with Vd serving as a surrogate marker for P-gp function. This model represents the optimal compromise between fit accuracy, precision, and biological plausibility [83].

This was demonstrated in a study conducted by Lee Y.J. et al. After intravenous administration of [^11^C]Verapamil, rapid distribution in the brain was observed for a short period, followed by slow elimination. In order to demonstrate the specific binding of the radiotracer to the P-gp site, two groups are compared by measuring the AUC_cereb_ and C_max cereb_; in fact, one of the groups is pre-treated with PSC833 and it shows a stark increase in the values of AUC_cereb_ and C_max cereb_ compared to the control group that was not pre-treated [85].

CsA also strongly influences the time-dependent brain uptake of [^11^C]Verapamil. The combined use of [^11^C]Verapamil and CsA, respectively, as a P-gp substrate and inhibitor, would be ideal for non-invasive measurement of P-gp transport across the human BBB [86]. The time–activity curves of [^11^C]Verapamil showed a peak 15 min after injection of [^11^C]Verapamil, both in the presence and absence of CsA. Both the PSC833 and CsA assays are useful to measure the kinetic profile of [^11^C]Verapamil as P-gp substrates in vivo at the BBB for quantitatively evaluating P-gp function at the BBB [69,87,88]. Verapamil shows largely linear PK in plasma and brain at both microdoses and therapeutic doses. It was also found that PET tracers administered at therapeutic doses are metabolized more slowly than at microdose levels [89]. Toornvliet R. et al. conducted a pilot study to assess the role of aging on P-gp function at the BBB using (R)-[^11^C]Verapamil and PET in healthy volunteers [90]. This pilot study shows that the Vd of the specific P-gp substrate (R)-[^11^C]Verapamil in the brain is higher in healthy elderly volunteers compared to healthy young volunteers. Therefore, during aging, the Vd of (R)-[^11^C]Verapamil in the brain increases, which may be associated with BBB dysfunction [91].

### 6.2. Fluorinated Verapamil Analogues

**Figure 4:**
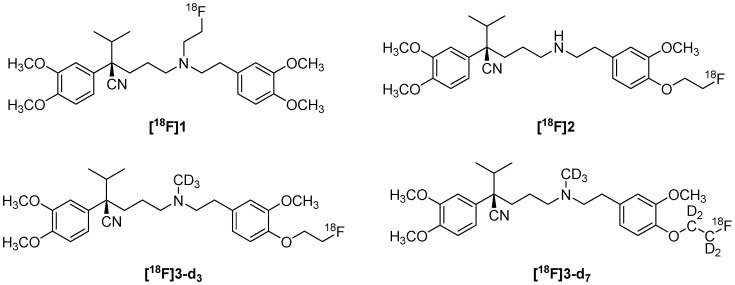
Structure of ^18^F-derivatives of verapamil [93,94].

One of the problems with [^11^C]Verapamil, which has led to its slow decline in use over the years, is the short half-life of 11C (20 min) [92]. For this reason, Raaphorst R.M. et al. developed fluorinated analogs such as (R)-*N*-[^18^F]fluoroethylverapamil ([^18^F]**1**) and (R)-*O*-[^18^F]fluoroethylnorverapamil ([^18^F]**2**,**Figure 4**), which could be radiolabeled with ^18^F. (R)-*O*-[^18^F]**2** is more specific for P-gp, despite its rapid metabolism, as well as a poor low metabolic stability [93]. Raaphorst R.M. et al. improved metabolic stability by incorporating deuterium into the tracer molecule. Additionally, greater metabolic stability was observed in methyl-containing analogs [^18^F]**3**-**d_3_** and [^18^F]**3-d_7_** (Figure 4), which could result from steric hindrance of enzymatic metabolism. [^18^F]**3-d_7_** thus showed similar in vivo behavior to (R)-[^11^C]Verapamil but with greater metabolic stability [94]. [^11^C]Verapamil and PET have been used for a long time in in vivo studies and human studies to investigate P-gp and the changes that substrates and inhibitors provoke on it [76,95,96].

### 6.3. Radiochemistry of (R)-[^11^C]Verapamil

**Figure 5:**
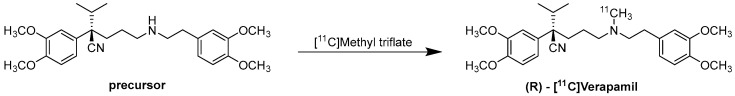
Synthesis of (R)-[^11^C]Verapamil. Precursor, [^11^C]Methyl triflate acetonitrile, 50 °C, 5 min [86].

Regarding the radiochemistry route, the reaction was performed with [^11^C]Methyl triflate in acetonitrile at 50 °C for 5 min (Figure 5) [86].

## 7. [^11^C]Loperamide and [^11^C]-*N*-Desmethyl-Loperamide

Loperamide and its metabolite *N*-desmethyl-loperamide are potent substrates of P-gp at the BBB, but [^11^C]-*N*-desmethyl-loperamide ([^11^C]dLop) is used as a PET radiotracer for imaging P-gp [97,98]. In a study conducted on human hepatic microsomes, it was observed that loperamide undergoes *N*-demethylation catalyzed by multiple cytochrome P450 isoforms, such as CYP2B6, CYP2C8, CYP2D6, and especially CYP3A4 [99]. The co-administration of ketoconazole (an inhibitor of CYP3A4) was partially effective in reducing the metabolism of [^11^C]Loperamide without showing any appreciable effect on the brain accumulation of [^11^C]dLop [100]. Zoghbi S.S. et al. demonstrated through in vivo and ex vivo studies conducted on wild-type and P-gp knockout mice that [^11^C]dLop is a better radiotracer than [^11^C]Loperamide for assessing P-gp function at the BBB, as the former has one fewer contaminating radiometabolite and thus better metabolic stability. Moreover, [^11^C]dLop presents [^11^C]CO_2_ as its final radiometabolite, which has minimal penetration into the brain [86,101,102]. dLop shows a pK_a_ of 7.3 [103]; therefore, it is a weak base. This allows for the passive diffusion and subsequent protonation of dLop in acidic environments, such as lysosomes, excluding mitochondria [104].

Liow-San Liowsulle J.-S. et al. analyzed the biodistribution of [^11^C]dLop in the whole body in monkeys. The highest accumulation of radioactivity was found in the liver, lungs, and kidneys. Despite the high accumulation in the kidneys, little radioactivity was excreted through urine. Instead, most of the radioactivity was likely excreted from the liver into the gastrointestinal tract. Following demethylation, [^11^C]dLop may be exhaled as [^11^C]CO_2_ [105]. Regarding plasma protein binding, no differences were found in that of [^11^C]-*N*-desmethyl-loperamide between mice and humans, unlike (R)-[^11^C]Verapamil, for which small but significant differences in the percentages of unbound (R)-[^11^C]Verapamil were observed [106]. Kannan P. et al. demonstrated that dLop acts as a P-gp inhibitor at high concentrations, whereas it behaves as a substrate at low concentrations, making the activity of dLop concentration-dependent. The in vivo results displayed that dLop is interesting only at low concentrations because it is a competitive substrate for P-gp [107].

Through a study conducted on WT and KO mice and WT mice pre-treated with cyclosporine, it emerged that the duration of the scan could play an important role in determining the kinetic parameters in the kinetic model. It has been demonstrated that 2 min after the administration of dLop, passive diffusion is the prevalent mechanism [108].

Farwell M.D. et al. studied P-gp activity with [^11^C]dLop in rat brains. These studies were carried out by pre-treating one group with CsA or Tariquidar (TQ) and leaving the other group untreated. CsA and TQ act as P-gp inhibitors; therefore, a higher uptake of [^11^C]dLop is observed [109]. The regional cerebral radioactivity uptake after intravenous administration of [^11^C]dLop, following various doses of DCPQ (0–16 mg/kg, iv), was measured in a monkey. It was found to increase almost linearly with the dose of DCPQ. This strongly indicates that [^11^C]dLop is sensitive to the degree of P-gp blockade in the brain. [^11^C]dLop has thus proven to be a strong substrate for brain P-gp in both mice and monkeys, as is [^11^C]Lop [103]. Kreisl W.C. et al. examined the ability of [^11^C]dLop to quantify P-gp function in humans after increasing doses of Tariquidar, highlighting that P-gp function can be quantified from a simple measure of brain uptake (AUC_10–30_) or from the brain influx rate constant (K1), both of which are strongly correlated. However, the single-pass extraction fraction (E) of [^11^C]dLop is sufficiently high that both AUC_10-30_ and K1 should be corrected for regional cerebral blood flow to separately measure the effects of permeability from those of drug administration [110,111]. The baseline AUC_10–30_ value found by Kreisl W.C. et al. [111] in humans was similar to the AUC_10–30_ value measured in baboons by Damont A. et al. (2.94 ± 0.66 SUV·min vs. 3.2 ± 0.5, respectively) [112]. The concomitant administration of the radiotracer allows for the use of lower doses of Tariquidar while producing a similar degree of P-gp inhibition. This method also reduces the amount of time needed to administer Tariquidar and perform the PET scan with [^11^C]dLop [113]. [^11^C]Lop has also been used to investigate P-gp modulation induced by antiepileptic drugs (AEDs) at the BBB, observing a small difference in [^11^C]dLop uptake after pretreatment with AEDs compared to treatment with cyclosporine [114]. In conclusion, [^11^C]-*N*-desmethyl-loperamide, like (R)-[^11^C]Verapamil, is not a highly sensitive radiopharmaceutical for detecting small (<50%) changes in P-gp expression/function at the BBB when used without the administration of a P-gp inhibitor [106].

### Radiochemistry of [^11^C]-N-Desmethyl-Loperamide

**Figure 6:**
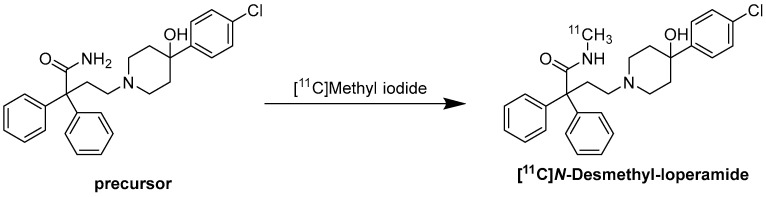
Synthesis of [^11^C]-*N*-desmethyl-loperamide. Precursor, [^11^C]Methyl iodide, KOH, DMSO, 80 °C, 5 min [86].

Regarding the radiochemistry route, the reaction was performed with [^11^C]Methyl iodide and KOH in DMSO at 80 °C for 5 min (Figure 6) [86].

## 8. [^11^C]Metoclopramide

Metoclopramide is an antiemetic and gastroprokinetic drug that acts as an antagonist of D2 receptors. [^11^C]Metoclopramide, on the other hand, is used as a PET radiotracer to measure P-gp function. It is a weak substrate of P-gp, showing significantly higher brain uptake under conditions of full P-gp functionality compared to previously described avid substrates such as [^11^C]Verapamil or [^11^C]dLop. Consequently, [^11^C]Metoclopramide has better sensitivity for detecting moderate changes in P-gp function at the BBB level compared to avid substrates [115]. Metoclopramide is metabolized by the CYP450 superfamily, particularly by the CYP3A4 and CYP2D6 isoforms. The main metabolites formed are M3 (a deethylation product) and M4 (a monooxygenation product), the latter being formed in the form of three different isomers. It has been shown that metoclopramide does not act as an inactivator of CYP2D6 [116]. However, Breuil L. et al. demonstrated that the induction of CYP with CBZ and the inhibition of CYP with RIT have a negligible impact on the plasma kinetics of [^11^C]Metoclopramide in rats. This supports the use of [^11^C]Metoclopramide as a PET probe for P-gp function at the BBB in situations where concomitant pharmacological treatment cannot be avoided [117]. The metabolism of metoclopramide can also be regulated independently of the intrinsic activity of metabolizing enzymes. Specifically, saturable transport mediated by a transporter on the sinusoidal plasma membrane of the liver was found to be essential for hepatic clearance, metabolism, and pharmacokinetics of metoclopramide [118]. Pottier G. et al. demonstrated, through studies on rats, that co-injecting [^11^C]Metoclopramide with non-labeled metoclopramide at its pharmacological dose (2 mg/kg) slows down the peripheral metabolism of the radiotracer while maintaining its ability to measure P-gp activity in the brain [119]. Alcohol exposure, on the other hand, had no impact on plasma concentrations, metabolism, protein binding, or brain kinetics of [^11^C]Metoclopramide [120]. From a kinetic perspective, Auvity S. et al. sought simplified parameters for the quantitative estimation of P-gp’s role in the brain kinetics of the radiotracer. In fact, AUC_30–60_ min was determined to be a better parameter that displays the impact of P-gp on the overall brain exposure to [^11^C]Metoclopramide without the need for an arterial input function [121]. The effect of an inhibitor on the kinetics of [^11^C]Metoclopramide was studied both in NHP and humans, using Tariquidar in the former and CsA in the latter. In both cases, P-gp inhibition led to a significant increase in the V_T_ of [^11^C]Metoclopramide. Pharmacokinetic modeling was performed in both cases to rationalize the V_T_ increase, using the 1-TCM model for NHP and the 1T2K model for humans. An increase in K1 (28.5% vs. 9%) and a concomitant decrease in K2 (36% vs. 15%) emerged [121,122]. Unlike avid P-gp substrates, [^11^C]Metoclopramide shows rapid and substantial initial brain uptake followed by rapid metabolism and elimination from plasma; therefore, the later part of the cerebral PET signal, which is mainly represented by unmetabolized [^11^C]Metoclopramide, allows for reliable calculation of k_E,brain_. The advantage of k_E,brain_ over cerebral V_T_, which is considered the gold standard for measuring P-gp function, is that it is calculated solely from dynamic cerebral PET data and does not require an arterial input function. However, k_E,brain_ is less sensitive than V_T_ in detecting maximum P-gp inhibition at the BBB [123]. The feasibility and relevance of k_E,brain_ of [^11^C]Metoclopramide have been evaluated in rats, NHP, and humans, showing that k_E,brain_ has adequate sensitivity to measure the impact of pharmacological inhibition of P-gp at the BBB. Given the substantial differences in BBB permeation and peripheral clearance of [^11^C]Metoclopramide between rats, NHP, and humans, k_E,brain_ values cannot be used to compare P-gp function across species. The main limitation of using k_E,brain_ is that this quantification method cannot be applied to other P-gp radiolabeled substrates that do not share the advantageous pharmacokinetic properties of [^11^C]Metoclopramide [123,124]. Bauer M. et al. studied in vivo the biodistribution of [^11^C]Metoclopramide in healthy males and females. The results demonstrated that the majority of the radiotracer was accumulated in the liver, with a minimal part of the radiotracer quickly excreted in urine, with very little difference between the male and female volunteers [125,126]. Apart from a slight accumulation in the basal ganglia (BG), the distribution across different brain regions was quite homogeneous; thus, no predominant accumulation was observed in regions rich in D2 dopamine receptors, such as the striatum [121,122]. In mice, it was demonstrated that the renal and hepatic uptake of [^11^C]Metoclopramide is partially mediated by transport through OCT1 and OCT2, while MATE transporters contributed to the urinary excretion of [^11^C]Metoclopramide and its radiolabeled metabolites [127]. As demonstrated by Bauer M. et al., [128] age-related differences in brain exposure to [^11^C]Metoclopramide were not caused by differences in the peripheral disposition of [^11^C]Metoclopramide, and reduced clearance of metoclopramide from the brain in elderly subjects may be related to an age-dependent decline in cerebral P-gp. Mairinger S. et al. evaluated in rats how pulmonary P-gp was affected by exposure to [^11^C]Metoclopramide (aerosolized model), showing a different behavior compared to other previously studied avid substrates such as (R)-[^11^C]Verapamil and [^11^C]dLop. Unlike (R)-[^11^C]Verapamil and [^11^C]dLop, the absence of P-gp activity led to an increase, rather than a decrease, in pulmonary exposure to [^11^C]Metoclopramide [127,128,129]. Over the years, [^11^C]Metoclopramide has also been used to assess both the immediate and delayed impact of BBB disruption induced by focused ultrasound with microbubbles [130]; furthermore, it was used to evaluate the effect of treatment with St. John’s Wort extract (SJW) with a high hyperforin content on central and peripheral P-gp activity, as well as peripheral CYP activity, in healthy volunteers [131].

### Radiochemistry of [^11^C]Metoclopramide

**Figure 7:**
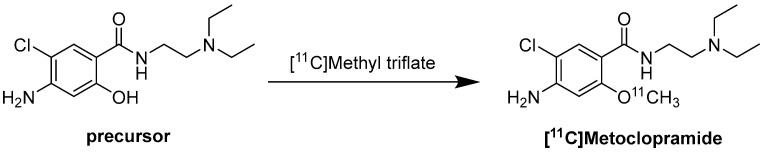
Synthesis of [^11^C]Metoclopramide. Precursor, [^11^C]Methyl triflate, NaOH 3N, acetone, 110 °C, 2 min [118].

Regarding the radiochemistry route, the reaction was performed with [^11^C]Methyl triflate and NaOH 3N in acetone at 110 °C for 2 min (Figure 7) [118].

## 9. [^18^F]MC225

[^18^F]MC225 is a weak P-gp substrate used as a PET tracer for imaging P-gp function in the BBB. This tracer exhibits higher baseline brain uptake, allowing for measurement of both increases and decreases in P-gp function. In vivo, [^18^F]MC225 has demonstrated good pharmacokinetic properties, a suitable signal-to-noise ratio, high sensitivity towards the target, and low levels of radiometabolites in the brain [132]. It is selective for P-gp, as additional inhibition of Bcrp had a negligible effect on the tracer’s brain uptake, as shown by Savolainen et al. [133]. The primary advantage of [^18^F]MC225 over [^11^C]Verapamil is its greater metabolic stability and lower number of metabolites in the brain. Over 96% of the parent radiopharmaceutical [^18^F]MC225 remained intact in the brain after 45 min, while radioactive metabolites constituted only 11–24% of total brain radioactivity at 60 min post-injection, compared to over 50% for [^11^C]Verapamil. The main metabolites of [^18^F]MC225 are products of defluoroethylation and demethylation. The [^18^F]fluoroethane/ethanol formed can likely enter the brain, but the fraction of metabolites observed in the brain after the PET scan was found to be small [134,135]. Garcia-Valera L. et al. studied different kinetic models of [^18^F]MC225 in NHP to assess P-gp function across varying scan durations. For short-duration scans, the 1-TCM model was preferred, with K1 being the best parameter for estimating P-gp function. For long-duration scans (>60 min), the 2-TCM model was preferred, where both K1 and V_T_ could be used to estimate P-gp function at the BBB [136]. [^18^F]MC225 has been compared to (R)-[^11^C]Verapamil to determine the best pharmacokinetic parameters to evaluate P-gp interactions for both radioligands. The main difference was the K2 value, which decreased for (R)-[^11^C]Verapamil, but remained unchanged for [^18^F]MC225, in the presence of an inhibitor. In addition, low baseline V_T_ values of (R)-[^11^C]Verapamil could hinder the quantification of increases in P-gp function, which are associated with further decreases in V_T_. This limitation is not present with [^18^F]MC225, making it suitable for measuring increases in P-gp function [136,137,138]. The difference in V_T_ between [^18^F]MC225 and [^11^C]Verapamil was also observed in a healthy human subject under both unblocked and blocked P-gp states, with blocking achieved via intravenous infusion of the specific P-gp inhibitor cyclosporine (2.5 mg/kg/h) starting 30 min before the scan. V_T_ in gray matter increased from 4.38 at baseline to 5.48 after cyclosporine administration, significantly higher than the V_T_ values previously reported for [^11^C]Verapamil (1.28 at baseline, 2.00 after P-gp inhibition), verifying [^18^F]MC225 as a promising tracer for measuring BBB P-gp function in humans [139,140]. The direct comparison between [^18^F]MC225 and (R)-[^11^C]Verapamil led to an initial evaluation of [^18^F]MC225 in human subjects as a Phase I study. Toyohara J. et al. validated the radiosynthesis process of [^18^F]MC225, assessing preclinical toxicity and radiation dosimetry based on distribution data in mice. Preclinical toxicology studies indicated that [^18^F]MC225 showed acceptable pharmacological safety at tracer concentrations required for adequate PET imaging. Regarding radiation dosimetry, a single intravenous injection of 185 MBq of [^18^F]MC225 resulted in an estimated effective dose of 2.9 mSv (with voiding 360 min post-injection) and 3.1 mSv (without voiding), well within the acceptable radiation dose limits for PET imaging. Cardiovascular toxicity studies further indicated that the cardiovascular activity of [^18^F]MC225 is negligible at tissue concentrations sufficient for in vivo quantification of P-gp activity [141,142]. In the Phase I trial conducted by Toyohara et al. with eight healthy subjects, [^18^F]MC225 was well tolerated, with no clinically significant adverse pharmacological effects or safety signals observed. Differences in V_T_ were noted across individuals in gray matter and choroid plexus regions, with interindividual variability (CV) in V_T_ in the 1-TCM, 2-TCM, and Logan plot models being 26% ± 4%, while intraindividual differences were relatively small (CV of V_T_ was 11% ± 3%). These large interindividual variances may be due to differences in P-gp density and/or function [143]. Mossel P. et al. analyzed the pharmacokinetics of [^18^F]MC225 in healthy volunteers, identifying the 2T4K model as the most accurate for describing its brain kinetics and quantifying P-gp function at the BBB with VB as the model parameter. However, [^18^F]MC225 displayed relatively large test retest (TRT) variability (28%) for V_T_, higher than the 9% reported for (R)-[^11^C]Verapamil [144]. [^18^F]MC225 has also been used to study circadian modulation of P-gp function in the brain. P-gp activity showed daily rhythms independent of sleep, suggesting that it is modulated by circadian rhythms in neurotransmitters, cytokines, or hormones [145]. Furthermore, [^18^F]MC225 was tested for its effects on anesthetic exposure in rats, showing significant increases in K2 values and corresponding decreases in V_T_ after prior anesthesia exposure, without significant changes in K1 [146].

### 9.1. Radiochemistry of [^18^F]MC225

**Figure 8:**
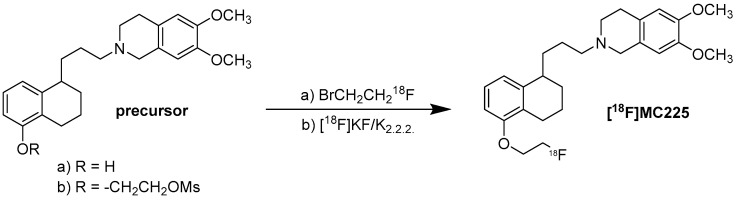
Synthesis of [^18^F]MC225. (a) Precursor, BrCH_2_CH_2_^18^F, NaH, DMF, 80 °C, 5 min [141]. (b) Precursor, [^18^F]KF/K_2.2.2_ complex, DMF, 140 °C, 30 min [132].

Regarding the radiochemistry route, two different approaches have been reported. The “two-step radiosynthesis” developed by Toyohara J. et al. involves the reaction of the phenol precursor with BrCH_2_CH_2_^18^F in the presence of NaH in DMF at 80 °C for 5 min [141]. In the second approach, a “one-step radiosynthesis” described by Garcia-Valera L. et al., the mesylate precursor is reacted with the [^18^F]KF/K_2.2.2_ complex in DMF at 140 °C for 30 min [132] (Figure 8).

### 9.2. Clinical Trials in Progress

Currently, [^18^F]MC225 is undergoing two different Phase II clinical trials: (i) [^18^F]MC225-PET in Neurodegenerative Disease, EudraCT Number: 2021-005024-37 (The Netherlands); (ii) The impact of gender differences in P-glycoprotein function measured with [^18^F]MC225 and PET, EudraCT Number: 2022-003664-25 (The Netherlands). Because several studies show an association between P-gp transporter overexpression and treatment-resistant depression [147], [^18^F]MC225 has received AIFA approval to begin an additional Phase II clinical trial investigating the correlation between P-gp and depression, and patient recruitment is currently ongoing.

## 10. [^11^C]-Labeled P-gp Inhibitors: Tariquidar, Elacridar, Laniquidar

**Figure 9:**
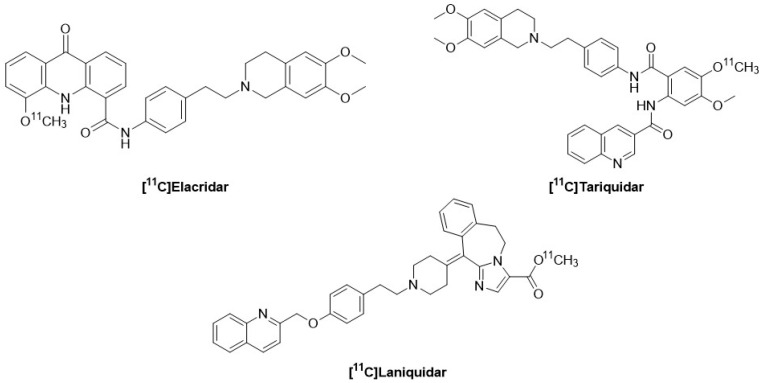
Structures of [^11^C] labeled P-gp inhibitors: Tariquidar, Elacridar, Laniquidar.

Tariquidar, Elacridar, and Laniquidar are third-generation inhibitors with high affinity for P-gp. They also cause fewer pharmacokinetic and pharmacodynamic interactions because they only minimally interfere with the cytochrome P-450 system [148]. Bauer F. et al. labeled Tariquidar with carbon-11 to evaluate its ability to measure P-gp function in rats. [^11^C]Tariquidar (Figure 9) does not exhibit selectivity towards P-gp as it also interacts specifically with BCRP at the BBB [149]. Dörner B. et al. labeled Elacridar with carbon-11, highlighting its ability to specifically bind to P-gp at the BBB. [^11^C]Elacridar (Figure 8), analyzed in vivo 2 h after the injection of unlabeled Elacridar, exhibited behavior comparable to (R)-[^11^C]Verapamil in a similar study setup [150]. The derivative [^18^F]Elacridar has also been developed, showing in vivo behavior similar to that of [^11^C]Elacridar. However, the significant degree of defluorination observed with [^18^F]Elacridar, along with low radiochemical yields, limited its subsequent use [151]. Compared to (R)-[^11^C]Verapamil, [^11^C]Elacridar and [^11^C]Tariquidar exhibited greater metabolic stability. The K1 values of [^11^C]Tariquidar and [^11^C]Elacridar were up to 10 times lower than those of (R)-[^11^C]Verapamil, suggesting that [^11^C]Tariquidar and [^11^C]Elacridar were more effectively effluxed at the BBB than (R)-[^11^C]Verapamil. The washout of [^11^C]Tariquidar and [^11^C]Elacridar from the brain was very slow [152]. The brain PET signal of [^11^C]Elacridar and [^11^C]Tariquidar was very low in rats, consistent with P-gp-mediated efflux transport. This limits the applicability of these tracers for visualizing brain P-gp density [153]. The tumor uptake of [^11^C]Tariquidar and [^11^C]Elacridar, on the other hand, demonstrated an absorption 2 to 3 times higher than the uptake in the brain, suggesting a better suitability of these radiotracers for tumor imaging rather than brain imaging [50]. The total body distribution of [^11^C]Elacridar and [^11^C]Tariquidar was studied in healthy men and women. They showed a high concentration of radioactivity in the liver, gallbladder, and intestines, with negligible concentration in the urinary bladder. This suggests that biliary excretion and possibly intestinal secretion are the primary elimination pathways for these radiotracers [154]. A marked decrease in radioactivity excreted in bile was also observed, in both humans and mice, when a pharmacological dose of unlabeled Tariquidar was co-administered with the radiotracer, suggesting inhibition of canalicular efflux transport activity [155]. Laniquidar was also labeled with [^11^C] by Luurtsema G. et al., but initial preclinical studies on this tracer did not show the expected brain uptake. They highlighted that at PET doses, [^11^C]Laniquidar (Figure 9) behaved as a substrate for P-gp rather than as an inhibitor [155]. [^11^C]Laniquidar has a K2 close to zero, suggesting that the best model was a single-tissue compartment model with a single parameter (K1), for which 5 min of data collection was sufficient. However, [^11^C]Laniquidar generates a high number of radioactive metabolites that cross the BBB; so, for the 60 min scan interval, it is better to use a kinetic model consisting of two parallel single-tissue compartment models, one for the parent [^11^C]Laniquidar and the other for the labeled metabolites [156]. The biodistribution of [^11^C]Laniquidar showed the highest uptake in the liver, followed by the spleen, kidneys, and lungs. In rats, the highest uptake was observed in the lungs, followed by the liver, spleen, and kidneys [157].

## 11. Conclusions

The sensitivity of PET probes in detecting moderate changes in P-gp function at the BBB has been shown to be different among clinically validated radiolabeled substrates. [^11^C]Verapamil was the first radiotracer used for PET imaging of P-gp because it is a selective substrate for it, thus allowing for non-invasive detection. [^11^C]Verapamil enables the study of P-gp function at the BBB, in normal conditions but also after treatment of inhibitors, and therapeutic drug interactions with P-gp. The racemic [^11^C]Verapamil has been replaced by its *R*-enantiomer since it has been shown that, despite being transported equally by P-gp, it has a slower metabolism [79]. However, [^11^C]Verapamil presents several disadvantages, including a rapid metabolism, which reduces the amount of available radiotracer in plasma, as well as being labeled with [^11^C], which is not preferred for clinical use. Due to these drawbacks, despite still being considered the “gold standard”, its use has progressively decreased in favor of better and more specific radiotracers. Among these are [^11^C]Lop and its metabolite [^11^C]dLop, which is a more specific substrate, as it does not interact with ion channels. The main advantage of [^11^C]dLop is its reduced metabolism [101]. Overall, however, [^11^C]dLop suffers from limitations similar to those of (R)-[^11^C]Verapamil, as the cerebral PET signal can be compromised by radiolabeled metabolites that are partially transported by P-gp. The baseline brain uptake of [^11^C]-*N*-desmethyl-loperamide is about two times lower than that of (R)-[^11^C]Verapamil, making it even more difficult to assess regional differences in cerebral P-gp function [90]. [^11^C]Metoclopramide, in contrast to [^11^C]Verapamil and [^11^C]dLop, is a weak substrate for P-gp, allowing better penetration through the BBB even when P-gp is active. It also has a more predictable metabolism, with its metabolites not significantly interfering with PET imaging. However, the PET radiotracer currently delivering the best results is [^18^F]MC225. Like [^11^C]Metoclopramide, it is a weak substrate, which is why it shows a higher uptake at the CNS level. Unlike the previously mentioned radiotracers, [^18^F]MC225 was designed as a specific substrate for P-gp, thus allowing greater specificity, sensitivity, and providing a more accurate and detailed image of P-gp activity, especially in the brain. [^18^F]MC225 also has reduced interaction with other transport proteins. Owing to these characteristics, [^18^F]MC225 is currently undergoing two different Phase II clinical trials. The table (Table 2) below provides a summary of the key advantages and disadvantages of the main radiotracers for P-gp.

## Figures and Tables

**Table 1:** Summary table of P-gp localization, expression (high ↑ or low ↓), its physiological and pharmacological roles in different body systems.

Organ/District	Localization	Physiological/Pharmacological Role
Intestine	Epithelial cells of the colon and ileum (↑); jejunum, duodenum, and stomach (↓)	Absorption and bioavailability; interacts with CYP3A4;
Liver	Bile ducts (↑)	Elimination of endogenous and xenobiotic metabolites
Kidneys	Proximal tubules (↑)	Excretion of endogenous and xenobiotic metabolites
CNS	Endothelial cells of BBB (↑)	Protects the CNS by limiting xenobiotic passage
Respiratory system	Ciliated epithelial cells, collecting ducts, lateral surfaces of serous cells in bronchial glands (↑); absent in goblet cells	Modulates pulmonary absorption of P-gp substrates (e.g., COPD drugs)
Placenta	Microvillous surface of syncytiotrophoblasts (↑)	Efflux of hydrophobic/cationic drugs into maternal blood; fetal protection
Heart	Arterioles and cardiac capillaries (↑)	Reduces intracellular drug concentration, lowering bioavailability

**Table 2:** Summary of function and key advantages and disadvantages for each listed P-gp radiotracers.

Tracer	Function	Advantages	Disadvantages
(R)-[^11^C]Verapamil	Avid substrate	“Gold standard” for comparing other P-gp ligands; extensive literature background and established models.	Poor sensitivity to moderate reductions in P-gp; strong dependence on metabolic correction; ^11^C-labeled.
[^11^C]dLop	Avid substrate	Very high dynamic range after P-gp blockade; strong increase in uptake with Tariquidar or other inhibitors; good selectivity for P-gp.	Low sensitivity to moderate decreases in P-gp function; presence of potential lipophilic metabolites; ^11^C-labeled.
[^11^C]Metoclopramide	Weak substrate	Metabolism present but manageable; ideal for detecting moderate pathophysiological changes; improved sensitivity for clinically relevant changes; validated in patients (epilepsy) without administering inhibitors.	Being a weak substrate, P-gp can limit both entry and increase efflux; there is still a need to standardize simplified outcomes; ^11^C-labeled.
[^18^F]MC225	Weak substrate	Longer half-life (easier logistics, multicenter possibilities, and longer scans); higher uptake than all previous tracers; sensitive to both down- and upregulation; reduced interaction with other proteins; good clinical translational potential (satisfactory initial evaluation in humans).	Studies are being carried out, and the radiotracer is being employed in Phase II clinical trials (in humans). Moreover, there are over 25 papers regarding this radiotracer.

## Data Availability

No new data were created or analyzed in this study. Data sharing is not applicable to this article.
